# Patients’ pathways to the emergency department: a scoping review

**DOI:** 10.1186/s12245-024-00638-w

**Published:** 2024-05-03

**Authors:** Målfrid Asheim Nummedal, Sarah Elizabeth King, Oddvar Uleberg, Sindre Andre Pedersen, Lars Petter Bjørnsen

**Affiliations:** 1 Trondheim Emergency Department Research Group (TEDRG), Department of Circulation and Medical Imaging, Faculty of Medicine and Health Sciences, Norwegian University of Science and Technology (NTNU), Trondheim, Norway; 2 Clinic of Emergency Medicine and Prehospital Care, St. Olav’s Hospital – Trondheim University Hospital, Trondheim, Norway; 3 Department of Research and Development, Division of Emergencies and Critical Care, Oslo University Hospital, Oslo, Norway; 4 Library Section for Research Support, Data and Analysis, The Medicine and Health Library, Norwegian University of Science and Technology (NTNU), Trondheim, Norway

## Abstract

**Background:**

Emergency department (ED) crowding is a common healthcare issue with multiple causes. One important knowledge area is understanding where patients arrived from and what care they received prior to ED admission. This information could be used to inform strategies to provide care for low acuity patients outside of the hospital and reduce unnecessary ED admissions. The aim of this scoping review was to provide a comprehensive overview of global published research examining the acute care trajectory of all ED patients.

**Methods:**

The scoping review was performed according to the JBI Manual for Evidence Synthesis and the PRISMA-SCR checklist. A comprehensive literature search was performed to identify studies describing where patients arrived from and/or whose pathway of care was before an ED visit. The search was conducted in MEDLINE, Embase, and the Cochrane Library from inception through December 5th, 2022. Two reviewers independently screened the records.

**Results:**

Out of the 6,465 records screened, 14 studies from Australia, Canada, Haiti, Norway, Sweden, Switzerland, Belgium, Indonesia, and the UK met the inclusion criteria. Four studies reported on where patients physically arrived from, ten reported how patients were transported, six reported who referred them, and six reported whether medical care or advice was sought prior to visiting an ED.

**Conclusion:**

This scoping review revealed a lack of studies describing patients’ pathways to the ED. However, studies from some countries indicate that a relatively large proportion of patients first seek care or guidance from a primary care physician (PCP) before visiting an ED. However, further research and published data are needed. To improve the situation, we recommend the development and implementation of a template for the uniform reporting of factors outside the ED, including where the patient journey began, which healthcare facilities they visited, who referred them to the ED, and how they arrived.

**Supplementary Information:**

The online version contains supplementary material available at 10.1186/s12245-024-00638-w.

## Background

Patient visits to emergency departments (EDs) around the world have significantly increased over the last few years [1–3]. This increase has led to ED crowding, which represents a mismatch between supply and demand [4]. Despite differences in national healthcare systems within Europe [5] and in the rest of the world, increased patient visits and ED crowding are common healthcare issues [1, 2]. Crowding of EDs represents a serious problem that leads to inappropriate and delayed treatment, increased length of stay (LOS), worse patient outcomes, and lower patient and staff satisfaction [3]. The causes behind the increase in patient volume are complex and likely due to a combination of several factors, including an ageing population [1]. Older patient groups often represent complex, multimorbid conditions requiring additional ED resources. Limited access to primary care and an increase in low-acuity patients have also been described as causes of crowding [1]. There is extensive literature on strategies to control patient demand [6], including greater access to primary care services [1], redirecting ambulances (ambulance diversion) [7], and filtering patients to alternative health care institutions [6]. These strategies show various levels of effectiveness across different countries and health systems.

A conceptual model used to describe crowding in the ED defines three major phases: input, throughput, and output [4]. Each phase has its own characteristics and means for managing patient logistics, although all the components need to be addressed to improve patient flow. A systematic review by Morley et al. concluded that the problem lies foremost outside of the ED and that the whole system of care should be included when identifying causes of crowding [1]. The input phase—together with output—involve factors contributing to ED crowding from outside the ED and includes any circumstance, occurrence, or system feature that affects the demand for ED services [4]. Patients can, for example, visit and receive different levels of care, be transported by the EMS, or be referred by a primary care physician (PCP) before entering the ED.

Healthcare systems across the globe offer different pathways for patient entry to the ED. The most prevalent are self-presentation and arrival via ambulance, with fewer countries using a strict referral system. More knowledge of patient trajectories within different healthcare systems, i.e., where ED patients arrived from and/or their pathways of care before an ED visit, is paramount to create targeted solutions for predicting and managing ED patient influx. Previous research on patient pathways has focused only on specific groups; cohorts referred by doctors to the ED (i.e., from the perspective of a general practitioner); patients who arrive by ambulance (i.e., from an emergency services perspective); or ED patients with specific chief complaints or symptoms (i.e., chest pain, abdominal pain, etc.). In contrast to this approach, we sought to include studies that described trajectories of the full spectrum of ED patients. The aim of this scoping review was to provide a comprehensive overview of what global published research has been conducted on patient trajectories to the ED.

## Methods

The scoping review was undertaken according to the principles presented in the JBI Manual for Evidence Synthesis [8] and followed the criteria set out in the PRISMA-ScR checklist [9]. The inclusion criteria for population, concept, and context (PCC) were defined. As per the protocol, studies that concerned the full spectrum of patients who arrived at an ED were eligible for inclusion; those that focused on selected groups (i.e., specific chief complaints, clinical findings, or demographics) were excluded. Eligible studies also had to describe one or both of the following:

1. where patients arrived from and/or where they were referred from (e.g., PCP, urgent care, outpatient clinic, self-referral, ambulance, or nursing home);
2. what actions or pathways they took before they visited the ED (i.e., how patients contacted or obtained acute health care services).

Observational/descriptive and registry studies from any country were eligible. Studies reported as abstracts were included if adequate data were provided.

The search was conducted in MEDLINE, Embase and the Cochrane Library from inception through December 5th, 2022, without language restrictions (SAP). The search was based on thesaurus- and free-text terms for the three main concepts ‘emergency medical services’, ‘prehospital’, and ‘descriptive patient data’ and adapted to the various databases (see Additional file 1 for a detailed description of the search strategies used in the databases). The resulting records were imported to EndNote, where duplicates were removed prior to the screening (SAP).

The titles and abstracts (first pass) and full papers that appeared relevant (second pass) were screened independently by two sets of reviewers (LPB and MAN or OU and SEK). For each set, half of the records were screened. Forward and backwards citation searches were also conducted for studies deemed relevant. The data were extracted by one of the reviewers and checked by another reviewer (MAN and SEK). Any disagreements were resolved by discussion among the review team until a consensus was reached.

## Results

**Fig. 1:**
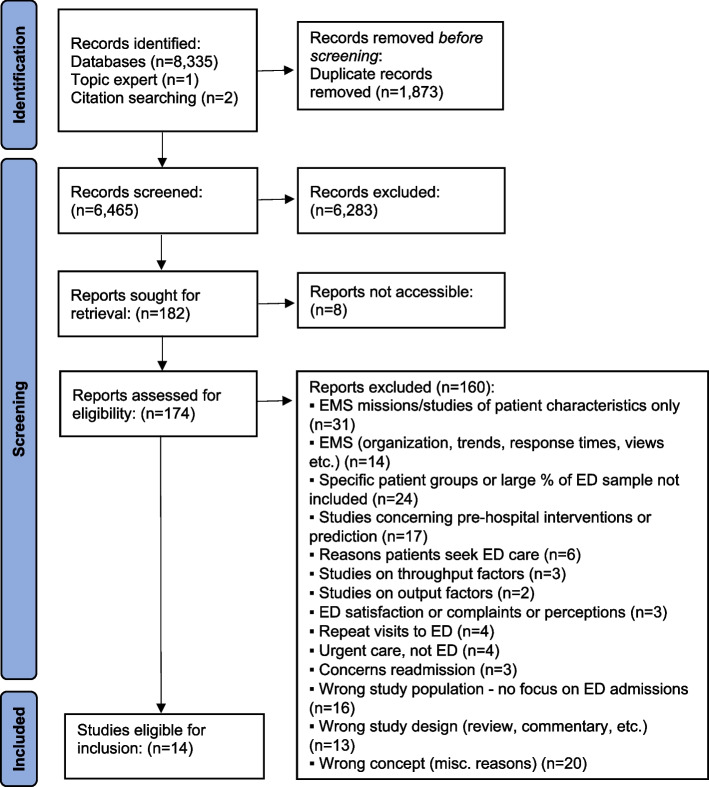
PRISMA flow diagram showing the inclusion and exclusion process. The figure shows the inclusion process and reasons for exclusion of identified records

In total, 6,465 titles and abstracts were screened, and 6,283 were deemed irrelevant to the topic area. Of the remaining 182 papers, 14 met the inclusion criteria (see Fig. 1 for PRISMA flow diagram [10]).

### Study characteristics

Four of the included studies were conducted in Australia [11–14], and two were conducted in both Canada [15, 16] and Sweden [17, 18]. One study each was conducted in Haiti [19], Norway [20], Switzerland [21], Belgium [22], Indonesia [23], and the UK [24]. The publication dates of the included studies spanned from 1993 to 2022, with all but three published in the last 10 years. Data were collected via surveys in seven of the studies and from databases in six; the remaining studies used both methods. The study and patient characteristics of the included studies are provided in Additional file 2.

An overview of the patient characteristics revealed that half of the studies included only adult patients. Adult patients were also most prevalent in the remaining studies [13–15, 19, 20, 22, 23]. The proportion of females ranged from 46 to 66% across the studies. The sample sizes were relatively large and ranged from 332 to 10,941,286. Differences between studies in terms of the amount and level of detail of the presenting conditions precluded a summary of these data. Four categories of information were commonly reported across the studies: 1) where patients physically arrived from, 2) how they were transported, 3) who referred them, and 4) whether medical care or advice was sought prior to visiting an ED. In all the categories, the evidence was limited by the small number of papers and by the sparseness of the data reported within them.

### Arrival origin sites

Knowing where patients physically arrived from contributes to understanding where demand most often begins. Only one study reported data that provided a full picture of where patients arrived from before visiting an ED [19] (Additional file 3). Although this Haitian study reflects a different social and healthcare context than that observed in Europe and North America, it shows that the highest proportion of patients (64%) arrived directly from their homes, with very few patients arriving from other places. This may also be the case in other countries, but published evidence is lacking. Three additional studies in Australia, Switzerland, and the UK reported low presentations of patients from nursing homes, ranging from 0.9% to 2% [11, 21, 24]. Additionally, one UK study with two data collection sites reported that 4.3% to 4.8% of patients were ‘referred’ from an office, shop, or workplace, and we assumed that they arrived at the ED directly from this place [24]. No further studies were found that directly addressed this topic.

### Mode of arrival

The mode of arrival or method of transport to an ED provides information about emergency service utilization as part of the patient journey. Ten studies reported this type of data [12–15, 17–20, 22, 23]. The proportion of patients who arrived by ambulance ranged from 8 to 43% (across 9 studies), with the majority arriving by public or private transport (Additional file 4). Very few patients arrived via police transport (0.5% to 0.9% across two studies). The mode of arrival was described as ‘self-presented’ or ‘walked-in’ in three studies [14, 20, 24], with proportions ranging between 69 and 91%; however, referral status or more specific means of transport were not reported. These data provide limited insight into the pathway of care prior to an ED visit for this group but may indirectly indicate the patients’ acuity.

### Referral patterns

The referral patterns indicate the last contact point before visiting an ED. Six studies (with seven data collection sites) variously reported on referrals through telephone services [17, 18, 22], urgent care centers [17, 20], outpatient clinics [17, 20], out-of-hours doctors [24], general practices [12, 17, 18, 22, 24], and the police. Five of these studies (with six data collection sites) also reported self-referral to the ED [12, 17, 18, 22, 24], and all the studies showed that this was the most prevalent mode of referral (ranging from 34 to 89%). The second most frequent points of referral were PCPs (ranging from 13 to 38% across four studies with five data collection sites), urgent care centers (ranging from 7 to 35% across two studies), and telephone services (0.5% to 11% across three studies) (Additional file 5).

**Fig. 2:**
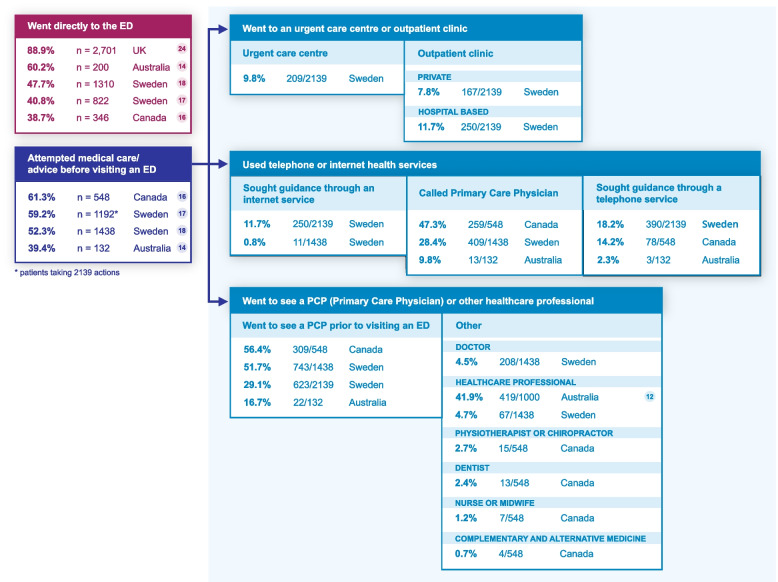
The proportion of patients who sought medical care or advice before visiting an ED. This figure provides an overview of the rates of direct referral to the emergency department (ED) and attempted care and advice provided before visiting an ED

Similar to the studies that reported referral rates, six studies specifically reported on the proportions of patients seeking medical care or advice before visiting an ED [12, 17, 18, 22, 24] (Fig. 2). However, it was sometimes unclear whether multiple sources of advice were sought for each patient and, if so, in what order and when. A relatively high proportion (up to 56%) of the patients seeking medical care did so through their PCP (physically or via a telephone call) or through another health professional rather than seeking advice using other options available (e.g., telephone or internet health service). The percentage of patients who went directly to the ED without seeking advice ranged from 39% (in Canada) to 89% (in the UK).

## Discussion

The scoping review revealed that there are very few published studies on patient trajectories prior to ED arrival and that detailed information regarding where ED patients are arriving and who referred them is mostly lacking. We attempted to include studies that represented the full spectrum of ED patients, but some of the included studies did not report on children or excluded some patients presenting for specialty care.

An informative finding in the included studies was the high proportion of patients seeking medical care or advice before visiting the ED. Based on a limited number of studies from four countries [14, 16–18], it appears that patients predominantly sought advice through their PCP. This initial evidence may help point to where some health service interventions could be considered. Potential solutions would, however, require further in-depth research with a design thinking approach that fully considers available resources. The important role of PCPs was acknowledged in the systematic review by Morley et al. [1], where limited access to primary care was presented as a possible factor contributing to ED crowding. Relatedly, there is a considerable difference in the proportion of patients who seek emergency care directly, with the UK having the highest proportion of patients seeking direct access (89%), followed by Australia (60%) and Sweden (41–48%). The reasons for these differences in self-referral rates are likely multifactorial, with a range of individual, cultural, and social factors playing a role in addition to different healthcare systems. For example, it is possible that differences in the availability and accessibility of primary care services contribute to the greater portion of patients accessing the ED directly in the UK and Australia. Sweden has a well-developed primary care system that may serve as an alternative to the ED and could explain why relatively fewer patients seek direct access to the ED in this country. However, the high proportion of low-acuity patients in Swedish EDs is also consistent with the idea that their primary healthcare system is under pressure [18].

Complimentary to the data on self-referral, the included studies show that large proportions of patients arrive at the ED by ambulance in Australia (28–43%) and Norway (38%), followed by Sweden (9–24%) and Canada (14–16%). Belgium reported a surprisingly low proportion of 9%, as well as Indonesia. The other study countries (Haiti, UK and Switzerland) did not explicitly report this information. While the data are very limited, it suggests higher ambulance usage in Scandinavia, compared to countries such as Haiti, Indonesia and some European countries. More exploration of the variations in arrival modes between countries would be useful to illustrate how different emergency health care services work across the globe.

Reliable comparisons of the scoping review data are difficult or impossible due to the lack of data, the inconsistency of the data, varying categorizations of the data, and varied use of terminology and definitions across the studies. In a report by Rowe and colleagues [25], the lack of uniform reporting from EDs is described as an underrecognized problem resulting in an inability to study the causes, characteristics, and results of ED crowding. As an example, such uniform reporting templates exist for out-of-hospital cardiac arrest and ED measurements for quality improvement [26]. Over time, the guidelines for reporting studies on out-of-hospital cardiac arrest became a crucial tool for assessing the relative strengths of various systems [27]. We believe it would be helpful to develop and implement a similar template for reporting factors outside the ED, such as the patient's mode of transportation, referral source, and healthcare facility. Such details would help us gain a greater understanding of ED usage. We will then be able to pinpoint areas where interventions could help manage, predict, and/or control patient flow.

Another main finding is the lack of studies presenting linked data between different levels of health services; only three of the included studies used data linkage: Arendts et al. 2012 [11], Carron et al. 2018 [21], and O’Loughlin et al. 2019 [12]. For future studies, the linkage between EMS and hospital data will provide the ability to track patients across health service levels and serve as a basis for describing complete patient trajectories [28].

### Limitations

This scoping review is limited by the small number of relevant studies identified. Studies where information on patient trajectories was not the primary objective and was only reported as an incidental finding may have been missed, as these would have been difficult to capture in our literature search. While studies that reported only on specific patient groups, e.g., chest pain or trauma, were excluded from this review, this was done to promote an ED-level perspective.

## Conclusions

Prior studies have shown varying levels of success regarding solutions for tackling ED crowding [29]. We need to explore every aspect of patient influx to find potential new strategies. One approach is to understand where the patients come from and which services they used before attending the ED. This scoping review revealed a lack of published studies and uniform reporting on patients’ pathways to the ED despite its importance. However, in several of the included studies, relatively large proportions of patients sought care or guidance from a primary care physician (PCP) prior to an ED visit in different countries. Further research and published data are needed on this topic, and we also recommend the development and implementation of a template for uniform reporting of factors outside the ED, including where their journey began, which healthcare facilities they visited, who referred them to the ED and how they arrived.

### Supplementary Information

**Additional file 1.** Description of the search strategies used in the databases

**Additional file 2.** Patient and study characteristics

**Additional file 3.** Overview of studies that reported where patients physically arrived from

**Additional file 4.** Patients’ mode of arrival or method of transport to an emergency department (ED)

**Additional file 5.** What health service or who referred patients to an emergency department (ED)

## Data Availability

No datasets were generated or analysed during the current study.

## Abbreviations

ED: Emergency department
LOS: Length of stay
PCP: Primary care physician
