# Supplementary material for: Patients’ pathways to the emergency department: a scoping review
Source: Int J Emerg Med. 2024 May 3;17:61. doi: 10.1186/s12245-024-00638-w (PMC11067175; doi:10.1186/s12245-024-00638-w)
Supplement: Supplementary file 1 — Additional file 1. Description of the search strategies used in the databases [file 12245_2024_638_MOESM1_ESM.docx]

**MEDLINE**

Ovid MEDLINE(R) and Epub Ahead of Print, In-Process, In-Data-Review & Other Non-Indexed Citations and Daily <1946 to December 5, 2022>

| # |  | Searches |
| --- | --- | --- |
| 1 |  | *Emergency Medical Services/ or ("emergency medical service?" or "emergency medical care" or "emergency unit?" or "emergency department?").ti,ab,kf. [Concept #1: Emergency medical services] |
| 2 |  | (prehospital or ((pre or prior or before or outside) adj2 (hospital? or "emergency department?" or ED or "emergency unit?" or "EU"))).ti,ab,kf. [Concept #2: Pre-hospital setting] |
| 3 |  | Statistics & Numerical Data.fs. or exp registry/ or (registry or registries or database? or statistic? or (patient? adj2 (data or records))).ti,ab,kf. [Concept #3: Descriptive patient data] |
| 4 |  | and/1-3 [Concept #1-3 combined] |

**Embase**

Embase <1974 to December 5, 2022>

| # | Searches |  |  |  |
| --- | --- | --- | --- | --- |
| 1 | *emergency health service/ or ("emergency medical service?" or "emergency medical care" or "emergency unit?" or "emergency department?").ti,ab,kw. [Concept #1: Emergency medical services] |  |  |  |
| 2 | (prehospital or ((pre or prior or before or outside) adj2 (hospital? or "emergency department?" or ED or "emergency unit?" or EU))).ti,ab,kw. [Concept #2: Pre-hospital setting] |  |  |  |
| 3 | exp register/ or (registry or registries or database? or statistic? or (patient? adj2 (data or records))).ti,ab,kw. [Concept #3: Descriptive patient data] |  |  |  |
| 4 | and/1-3 [Concept #1-3 combined] |  |  |  |
