# Supplementary material for: Patients’ pathways to the emergency department: a scoping review
Source: Int J Emerg Med. 2024 May 3;17:61. doi: 10.1186/s12245-024-00638-w (PMC11067175; doi:10.1186/s12245-024-00638-w)
Supplement: Supplementary file 2 — Additional file 2. Patient and study characteristics [file 12245_2024_638_MOESM2_ESM.docx]

| Reference  Study country | Place of data collection and setting | Study design (as described by authors) and data collection method  Date of data collection | Study objective (verbatim) | Study inclusion criteria | Number of patients included in the analysis | Age (years) | Gender  (% female) | Conditions (very broadly) |
| --- | --- | --- | --- | --- | --- | --- | --- | --- |
| Aluisio et al., 2014 (19)    Haiti | 1 hospital ED (Fort Liberté Hospital); low income setting which lacks prehospital services | Observational, cross-sectional study using a questionnaire    April 2, 2012 to June 5, 2012 | To assess prehospital characteristics in the North East Department with the aim of providing baseline data to inform prehospital systems development. | Patients presenting for non-specialty care (treated in either the Ambulatory Care Clinic or the Emergency Care Department) | 441 patient encounters, with 145 (32.9%) from the Emergency Care Department and 296 (67.1%) from the Ambulatory Care Clinic | Median 24.9 years  < 5 years: 87 (19.7%)  ≥5 to <45 years: 260 (64.4%) | 266 (62.0%) | Trauma patients accounted for 24.7% of the population sampled, while patients presenting for other medical concerns comprised the remainder |
| Arendts et al., 2012 (11)    Australia | 6 hospital EDs; urban and rural hospitals | Descriptive; individual patient data obtained from merged databases    Starting 1 July 2006 for 12 months | To describe the clinical profile and outcomes of patients presenting to ED from RACF in one Australian state (New South Wales (NSW)). | People aged 65 years and over and living in residential aged care facilities | 4,680 | Median 85 years | 2,976 (63.6%) | Neurological 434 (9.4%)  Cardiovascular 630 (13.6%)  Gastrointestinal 497 (10.8%)  Respiratory 631 (13.7%)  Skin 180 (3.9%)  Urinary 317 (6.9%)  Orthopaedic 308 (6.7%)  Other surgical 93 (2.0%)  Psychiatric 143 (3.1%)  Other medical 505 (10.9%)  Minor trauma 645 (14.0%)  Catheter/device problem 108 (2.3%)  Not categorised 78 (1.7%) |
| Bjørnsen et al., 2013 (20)    Norway | 1 hospital (St. Olav’s University Hospital); serves the city of Trondheim and is the local hospital for the 280,000 inhabitants in  Soer-Troendelag County | Descriptive, retrospective analysis; data collected from one database  December 1, 2010 to December 1, 2011 | To describe the epidemiology of patients at an urban Norwegian university hospital ED and examine the variations in patient gender and age, timing of visits, acuity level of the presenting complaints, and disposition. | All patients presenting at the ED | 21,755 | <16 years: 1.2%  16-24: 7.1%  25-44: 16.9%  45-64: 25.2%  >65: 49.7% | 10,812 (49.7%) | Specific conditions not reported; medical patients (66%), surgery patients (30%), and other (gynaecology, paediatrics, ear-nose-throat, and neurosurgery) (4%) |
| Brasseur et al.,  2021 (22)  Belgium | 2 hospitals; two facilities of the University Hospital of Liège, the Notre Dame des Bruyères (CHUNDB) site and the Sart Tilman (CHUST) site. Suburban and urban. | Survey using a questionnaire  9 consecutive days in March, 2017 | To determine the drivers and rationale motivating walk-in patients to reach the ED rather than use primary care resources. | All patients (adults and children) who presented on their own to the Emergency Department. Every patient referred to the Emergency Department by a primary care physician or a telephone triage nurse. Every patient referred to the Emergency Department after calling the European Emergency Number (either by ambulance, paramedical intervention team or medical transport) | 1,945 | Mean 39.8 ± 24.55 years | 975 (50.1%) | NR |
| Brice et al., 2022 (23)  Indonesia | 5 hospitals; major hospitals in Jakarta. These five hospitals comprised four government-funded and one private hospital. | Cross-sectional survey using a questionnaire  December, 2019 | The primary objectives were to answer the following questions: 1) How do patients currently reach the hospital and does transportation mode differ by emergency type? 2) How do ambulances perform, in terms of measures such as response time or time to treatment, as compared with other modes of transport in Indonesia? | Patients who attended the hospital EDs during December 2019. Parents and guardians represented patients under 18 years of age. | 1,964 | 44 years (IQR: 26 to 58) | 949 (48.3%) | Medical 1236 (62.9%)  Respiratory 301 (15.3%) Trauma 169 (8.6%)  Cardiovascular 130 (6.6%)  Neurological 72 (3.7%)  Other 56 (2.9%) |
| Carron et al., 2017 (21)    Switzerland | 1 hospital (Lausanne University Hospital); a public university hospital that provides primary care for 300,000 inhabitants as well as tertiary care for Western Switzerland | Descriptive, retrospective analysis; a database was created from two administrative databases (an information database and a patient’s flow database)    January 1, 2005 to December 31, 2010 | To investigate the evolution over time of the number of ED visits by nursing home residents in a Swiss academic medical center, and to describe these ED visits (i.e., mode of admission, schedule, triage level, main reason for visit), as well as their outcomes (i.e., length of ED and hospital stay, ED and hospital discharge dispositions). | All nursing home residents aged 65 years and over who visited the ED from year 2005 to 2010 | 3,590 | Mean 84.3 years (SD 7.8) | 2,353 (65.5%) | Injury (32.0%)  Respiratory (12.9%)  Cardio (11.9%)  Gastro (9.6%)  Neuro (8.8%)  Others (24.8%) |
| Göransson et al., 2013 (17)    Sweden | 1 hospital; a level one trauma centre at a university hospital with 70,000 patient visits | Prospective descriptive study using a questionnaire    12 weeks in 2008 | To investigate whether patients come directly to the ED or whether they have taken any other actions or activities within the healthcare system before attending the ED. | Adult ambulatory and patients arriving to the ED by ambulance | 2,014 | Median 46 years (range 18-98) | 1,099 (54.6%) | A bar graph in the paper presents information on patients’ chief complaints, grouped according to the tenth revision of the International Statistical Classification of Diseases and related health problems (ICD-10) |
| Han et al., 2007 (16)    Canada | 2 hospitals (University of Alberta Hospital and the Royal Alexandra Hospital) from urban sites | Cross-sectional survey using a questionnaire    10-week period from September 2004 to November 2004 | The primary objective was to examine the frequency and determinants of patients’ efforts to access alternative care before ED presentation in 2 tertiary hospitals in the Capital Health Region of Alberta. | Patients aged 17 years and older presenting to the ED but not requiring  resuscitation | 894 | Mean 44.1 years (SD 19.7) | 456 (51%) | Injury presentation: 192 (22%) |
| Henricson et al., 2022 (18)  Sweden | 43 hospitals; 43 of Sweden’s 72 EDs | Cross-sectional study. Each ED filled in a form to register the pathway leading each adult patient to the ED. Supplemental data on all registered patients were acquired from electronic health records  24 h on April 25th, 2018 | To provide a national overview of pathways, degree of medical acuteness according to triage, chief complaints, and hospital admission rates for adult patients (≥18 years) visiting Swedish EDs during 24 h. | All adult patients (18 years or above) attending Swedish EDs during 24 h | 3,875 | Median 59 years, (range 18 to 107) | 1,938 (50%) | The most common chief complaints at ED presentation were abdominal pain (524; 14%), followed by chest pain (369; 10%) and breathing problems (278; 7%) |
| Jankowski et al., 1993 (24)    UK | 2 hospitals; one in inner London and one outside London | Survey study using a questionnaire    First two weeks in December (in London) and  last two weeks of January (outside London) [year not stated] | To compare the sociodemographic characteristics, diagnoses, and mode of referral of people and emergency admissions between an accident and emergency department in inner London and one in a town outside London. | Adults newly attending each accident and emergency department | 3,039 (1,476 in inner London and 1,563 outside London) | Over 65 years: 269 (18.2%) in London and 279 (17.9%) outside of London | Not reported | Diagnostic categories (inner and outer London):  Circulatory 62 (4.7%) and 62 (4.5%)   Gastrointestinal 104 (8%) and 63 (4.6%)   Respiratory 86 (6.5%) and 65 (4.7%)   Musculoskeletal 316 (24%) and 457 (33%)   General (non-specific) 86 (6.5%) and 68 (4.9%)   Skin (including minor trauma) 358 (27.2%) and 437 (31.6%)   Urological 46 (3.5%) and 26 (1.9%)   Gynaecological 65 (4.9%) and 19 (1.4%)   Neurological 55 (4.2%) and 54 (3.9%)   Blood disorders 9 (0.7%) and 2 (0.1%)   Psychological 37 (2.8%) and 11 (0.8%)   Endocrine or metabolic 9 (0.7%) and 11 (0.8%)   Other 35 (2.7%) and 63 (4.6%)   Uncertain of diagnosis 22 (1.7%) and 20 (1.4%)   Unknown 27 (2.1%) and 26 (1.9%) |
| O'Loughlin et al., 2019 (12)    Australia | 1 hospital ED (Cairns Hospital) which is a large, public, tertiary referral hospital in far north Queensland | Cross-sectional survey using a patient experience survey and linked databases    13 March to 11 April 2014 | To explore factors that influence presentation at a regional hospital ED and identify opportunities to reduce attendance, particularly for adults with chronic conditions. | People attending the ED of a large, public hospital who were 18 years or older | 3,229 (with survey data available for 1000 patients) | Mean 47.1 years (SD 19.2) | 1,587 (49.2%) | Reasons for presentation to the ED are only presented in a figure; the authors stated that the largest categories were for injury, poisoning and other harm |
| Pryce et al., 2021 (13)    Australia | 1 hospital (Launceston General Hospital) providing ED services for approximately 145,000 people | Retrospective analysis of two-years ED presentation data, prospective observational analysis and a focus group analysis    Retrospective part of study (i.e. assessing input in the ED): 2016 and 2017 | To identify input, throughput and output factors contributing to ED patient flow bottlenecks and extended ED length of stay (EDLOS). | ED presentations were included [no other details were reported] | 89,013 ED presentations | Median 40.0 years (range 0-105) | Not reported | Not reported |
| Robinson et al., 2015 (14)    Australia | 2 hospitals (Royal Adelaide Hospital and The Queen Elizabeth Hospital); two adult metropolitan EDs in South Australia | Prospective observational study using a questionnaire    48 hours | To determine patients’ points of contact prior to decision making processes before presenting to an Emergency Department for treatment. To obtain data that may inform future exploration of targeted Emergency Department avoidance strategies. | All patients who presented during the 24 h period | 332 | 0—18: 17 (5.1%)  19—36: 107 (32.2%) 37—50: 59 (17.8%)   51—70: 78 (23.5%)  71+: 71 (21.4%) | 151 (45.5%) | The major presenting complaint across both sites were ‘single trauma’ (n=82, 21%) followed by psychosocial problems (n=48, 14.5%) |
| Strum et al., 2022 (15)  Canada | All EDs in one province (Ontario) | Population-level retrospective cohort study  Between January 1, 2010 and December 31, 2019 | To describe changes in the incidence of paramedic transport to emergency departments in Ontario. Specifically, to examine changes in population growth against changes in ED visitation by paramedic transport and walk-in, as well as to define and describe trends in annual paramedic utilization for transport to the ED. | All patients triaged in an Ontario ED and arrived by either ambulance or walk-in between January 1, 2010 and December 31, 2019 were included. | 10,941,286 |  | NR for full population | NR |
