# Supplementary material for: Patients’ pathways to the emergency department: a scoping review
Source: Int J Emerg Med. 2024 May 3;17:61. doi: 10.1186/s12245-024-00638-w (PMC11067175; doi:10.1186/s12245-024-00638-w)
Supplement: Supplementary file 3 — Additional file 3. Overview of studies that reported where patients physically arrived from [file 12245_2024_638_MOESM3_ESM.docx]

| Reference |  | Where patients physically originated from before visiting the ED  % (n) | | | | | |
| --- | --- | --- | --- | --- | --- | --- | --- |
|  | **Home** | **Office, shop, or workplace** | **School** | **Road/Street** | **Market** | **Nursing home** | **Other** |
| Jankowski et al., 1993 (25)  UK*, Inner London |  | 4.3 (64) |  |  |  | 1.4 (20) |  |
| Jankowski et al. (25), 1993  UK*, Outer London |  | 4.8 (75) |  |  |  | 0.9 (14) |  |
| Arendts et al., 2012 (12)  Australia |  |  |  |  |  | 2.0** |  |
| Aluisio et al., 2014 (2)  Haiti | 63.9 (147) | 8.7 (20)^1^ | 5.7 (13) | 16.1 (37) | 2.2 (5) |  | 3.5 (8) |
| Carron et al., 2017 (22)  Switzerland |  |  |  |  |  | 1.8** |  |
| All studies, range | 63.9 | 4.3 - 8.7 | 5.7 | 16.1 | 2.2 | 0.9- 2.0 | 3.5 |

*Data were described by Jankowski et al. 1993 as ‘mode of referral’. An assumption was made that nursing home patients directly arrived at the ED from this place

**These figures refer to the proportion of patients coming from nursing homes out of all ED presentations (proportions from other places are not presented)

^1^ Work
