# Supplementary material for: Patients’ pathways to the emergency department: a scoping review
Source: Int J Emerg Med. 2024 May 3;17:61. doi: 10.1186/s12245-024-00638-w (PMC11067175; doi:10.1186/s12245-024-00638-w)
Supplement: Supplementary file 4 — Additional file 4. Patients’ mode of arrival or method of transport to an emergency department (ED) [file 12245_2024_638_MOESM4_ESM.docx]

| Reference  Study country |  | Patients’ mode of arrival or method of transport to the ED – % out of total ED population | | | | | | |
| --- | --- | --- | --- | --- | --- | --- | --- | --- |
|  | **Self-present**  **% (n)*** | **Ambulance**  **% (n)** | **Public  transport**  **% (n)** | **Personal vehicle**  **% (n)** | **Walked**  **% (n)** | **Bicycled**  **% (n)** | **Police**  **% (n)** | **Other**  **% (n)** |
| Aluisio et al., 2014 (20)  Haiti |  |  | 68.3 (300)^1^ | 5.2 (23) | 17.1 (75) | 1.4 (6) | 0.5 (2) |  |
| Bjørnsen et al., 2013 ** (21)  Norway |  | 38 (5,518)^2^ | 5 (757)^3^ |  |  |  |  |  |
| Brasseur et al., 2021 (23)  Belgium |  | 8.8 (171)^4^ |  | 91.2 (1774) |  |  |  |  |
| Brice et al., 2022 (24)  Indonesia |  | 9.3 (182) | 33.5 (659)^5^ | 50.0 (983)^6^ |  |  |  | 3.8 (75) |
| Göransson et al., 2013  Sweden | 90.9 (1,832) | 8.1 (163)^7^ |  |  |  |  |  | 0.8 (16)^8^ |
| Henricson et al., 2022 (19)  Sweden |  | 23.7 (920) |  |  |  |  |  |  |
| O'Loughlin et al., 2019 (12) Australia |  | 42.9 (1,385) |  |  |  |  |  |  |
| Pryce et al., 2021 (13)  Australia | 69.3 (61,642) | 27.9 (24,845) |  |  |  |  | 0.9 (830) | 1.9 (1,695) |
| Robinson et al., 2015 (14)  Australia |  | 40.7 (135) | 18.0 (60) | 41.3 (137) |  |  |  |  |
| Strum et al., 2022 (15)  Canada | 2010: 86.3 (4,357,670)  2019: 83.8  (4,940,524) | 2010: 13.7 (698,479)^9^  2019: 16.2 (953,613)^9^ |  |  |  |  |  |  |
| All studies, range | 69.3 - 90.9 | 8.1 - 42.9 | 5 - 68.3 | 5.2 - 91.2 | 17.1 | 1.4 | 0.5 -0.9 | 0.8 - 3.8 |

*In these studies, the arrival mode was reported as “self-present” or “walk-in”, but transport details were not specified.

**In this study, the remaining % of patients arrived via transfer from other areas within the hospital. The total ED population within the data collection period is not reported.

Motorcycle taxi or public vehicle

^2^ Also includes air ambulance

^3^ Reported as own transportation, which is presumed to be by taxi or private car

^4^ Reported as help from rescue services

^5^ Ride sharing service car or taxi

^6^ Car or motorcycle

^7^ This study reported ambulance [road or helicopter] and police together (information obtained from the author)

^8^ A non-urgent ambulance

^9^ Paramedic transport
