# Supplementary material for: Patients’ pathways to the emergency department: a scoping review
Source: Int J Emerg Med. 2024 May 3;17:61. doi: 10.1186/s12245-024-00638-w (PMC11067175; doi:10.1186/s12245-024-00638-w)
Supplement: Supplementary file 5 — Additional file 5. What health service or who referred patients to an emergency department (ED) [file 12245_2024_638_MOESM5_ESM.docx]

| Reference  Study country | What health service, or who, referred patients to an ED | | | | | | | | | |
| --- | --- | --- | --- | --- | --- | --- | --- | --- | --- | --- |
|  | **Self-referral (or family/friend)  % (n)** | **Telephone or internet service**  **% (n)** | **Urgent care centre   % (n)** | **Outpatient**  **clinics  % (n)** | **Out-of-hours doctor**  **% (n)** | **PCP**  **% (n)** | **Informal referral from the general practice**  **% (n)** | **Police % (n)** | **Other**  **% (n)** | **Not known**  **% (n)** |
| Bjørnsen  et al., 2013 (20)  Norway |  |  | 35 (5,176) | 3 (365)^1^ |  |  |  |  |  |  |
| Brasseur et al., 2021 (22)  Belgium | 68.2 (1,326) | 6.6 (129) |  |  |  | 16.6 (323) |  |  | 8.6 (167)^2^ |  |
| Göransson  et al., 2013 (17)  Sweden* |  | 11.0 (221)^3^ | 7.1 (142) | 6.2 (124)^4^    15.2 (181)^5^ |  | 22.2 (448) |  |  |  |  |
| Henricson  et al., 2022 (18)  Sweden | 33.8 (1,310) | 0.05 (2)^6^ |  |  |  | 12.8 (497) |  |  | 3.4 (130)^7^ |  |
| Jankowski  et al., 1993 (24)  UK, Inner London | 66.7 (984) |  |  |  | 0.9 (14) | 12.4 (183)^8^  0.3 (5)^9^  Total: 12.7 | 1.9 (28) | 1.2 (18) | 4.7 (69) | 6.2 (91) |
| Jankowski  et al., 1993 (24)  UK, Outer London | 67.7 (1,058) |  |  |  | 1.2 (19) | 14.7 (229)^8^  1.2 (18)^9^  Total: 15.9 | 2.7 (42) | 1.2 (18) | 4.0 (63) | 1.7 (27) |
| O'Loughlin et al., 2019 (12)  Australia | 88.6 (2,860)^10^ |  |  |  |  | 29.0 (290)** |  |  |  |  |
| Robinson et al., 2015 (14)  Australia | 60.2 (200) |  |  |  |  |  |  |  |  |  |
| All studies, range | 33.8 - 88.6 | 0.05 - 11.0 | 7.1 - 35 | 3 - 15.2 | 0.9 - 1.2 | 12.7 - 37.6 | 1.9 - 2.7 | 1.2 | 4.0 - 8.6 | 1.7 - 6.2 |

*Percentages of referrals have been re-calculated using the total sample as the denominator

**Based on a subset of patients surveyed (n=1000)

Outpatient clinics or transferred

^2^ Reported as hospital specialist or third person

^3^ The Stockholm Care Guide through the telephone [service]

^4^ Private clinic

^5^ Hospital clinic

^6^ Internet medical service

^7^ Other doctor or healthcare provider

^8^ Patients’ own GP

^9^ Private GP or clinic

^10^ Self/family/friend
